# AI-enabled precision prediction and proactive management of cutaneous toxicities in cancer immunoradiotherapy (ICI+RT)

**DOI:** 10.3389/fonc.2026.1800254

**Published:** 2026-04-02

**Authors:** Huizhan Jia, Shuixia Liu, Jungang Ma

**Affiliations:** Department of Cancer Center, Daping Hospital, Army Medical University, Chongqing, China

**Keywords:** artificial intelligence, cutaneous toxicity, dose topology, immune-related adverse events, immunoradiotherapy, precision toxicity management, radiation dermatitis, radiomics

## Abstract

Immunoradiotherapy has become an increasingly important strategy for the treatment of advanced malignant tumors, but its broader application is accompanied by a high incidence of cutaneous toxicities, including radiation dermatitis and immune-related skin adverse events. These toxicities often emerge early during treatment, exhibit substantial inter-patient heterogeneity, and can compromise treatment continuity and patient quality of life. Conventional management remains largely reactive and grading-based, offering limited capacity for individualized risk assessment or early intervention. Recent advances highlight that cutaneous toxicity in immunoradiotherapy arises from the convergence of radiation-induced tissue injury and immune checkpoint blockade–driven immune amplification, involving interconnected pathways such as DNA damage–associated danger signaling, innate immune activation, cytokine amplification, and dysregulated T-cell effector responses. This biological complexity limits the predictive utility of single-factor or mechanism-isolated approaches, underscoring the need for integrative, data-driven strategies. In this mini-review, we synthesize emerging AI-enabled approaches for precision prediction and management of cutaneous toxicities in immunoradiotherapy. We focus on how clinicodosimetric variables, spatial dose topology, imaging- and radiomics-derived tissue susceptibility, and accessible immune–inflammatory surrogates can be integrated into pathway-informed predictive models. We further discuss translational frameworks that embed prediction into clinical workflows, enabling plan-aware exposure mitigation, proactive supportive care stratification, and dynamic on-treatment risk updating. Collectively, these advances position cutaneous toxicity as a tractable and clinically meaningful endpoint for precision management in immunoradiotherapy, aligned with the goals of data-driven oncology.

## 1 Introduction

The integration of radiotherapy with immune-based treatments has reshaped the therapeutic landscape for advanced malignant tumors (1). Beyond its cytotoxic effects, radiotherapy is now recognized as a potent immunomodulatory intervention capable of enhancing antigen presentation, promoting immune cell infiltration, and synergizing with immune checkpoint blockade (2, 3). Immunoradiotherapy refers to the combination of radiotherapy and immune checkpoint inhibition (ICI+RT). This paradigm of immunoradiotherapy has demonstrated encouraging clinical activity across multiple tumor types, particularly in settings where durable local and systemic control remain difficult to achieve.

However, the expanding use of immunoradiotherapy has been accompanied by an increased burden of treatment-related toxicities, among which cutaneous adverse reactions are among the most frequent and clinically visible (4). Radiation-induced dermatitis and immune-related skin toxicities often occur early during treatment, vary widely in severity, and substantially affect treatment continuity, patient quality of life, and supportive care demands (5, 6). Importantly, when radiotherapy is combined with immune checkpoint inhibitors, skin toxicities may exhibit atypical presentations, prolonged courses, or enhanced severity, reflecting overlapping tissue injury and immune activation processes (7).

Despite their clinical relevance, cutaneous toxicities in immunoradiotherapy remain largely managed through empirical assessment and reactive intervention (8). Current grading systems capture severity retrospectively but provide limited capacity for individualized risk prediction prior to treatment initiation (9). Mechanism-based explanations alone are insufficient to account for the marked inter-patient heterogeneity observed in clinical practice, where similar treatment regimens can result in vastly different skin responses (10, 11). This gap underscores the need for predictive strategies that move beyond single-factor reasoning and toward integrative, patient-specific modeling.

**Figure 1:**
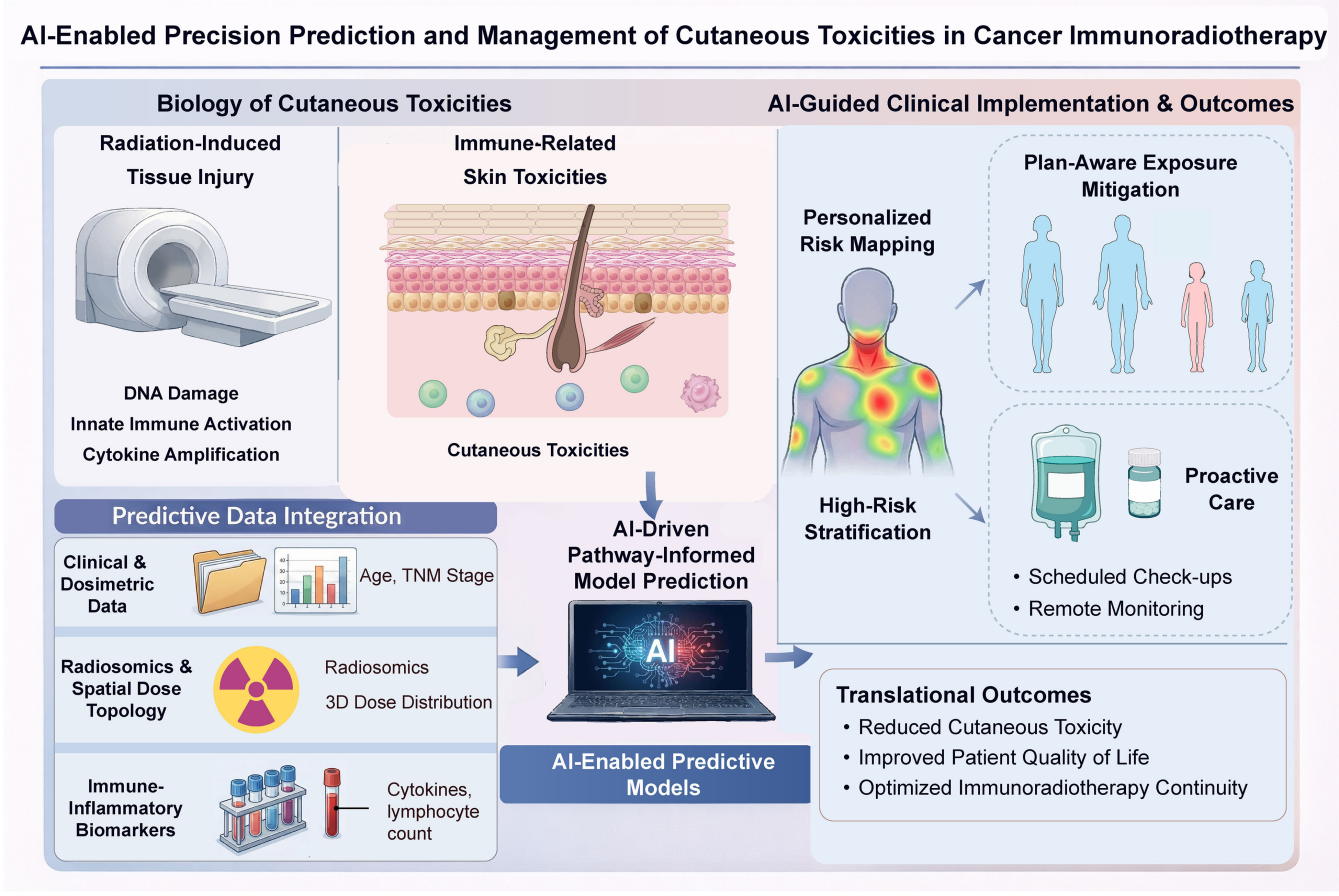
AI-enabled precision framework for predicting and managing cutaneous toxicities in cancer immunoradiotherapy. Multimodal clinical, dosimetric, imaging, and immune–inflammatory data are integrated into pathway-informed AI models to enable individualized risk stratification, plan-aware exposure mitigation, and proactive supportive care.

In this context, data-driven and artificial intelligence (AI)–based approaches offer a compelling opportunity to enable precision toxicity management in immunoradiotherapy (12). By integrating clinical variables, treatment parameters, imaging features, and emerging biological indicators, AI-enabled models have the potential to identify patients at high risk for cutaneous toxicities, inform proactive mitigation strategies, and support individualized treatment adaptation (13, 14). Prediction frameworks increasingly distinguish baseline risk estimation, severity stratification, and on-treatment risk updating, while clinical use depends on rigorous external validation and calibration. The following sections synthesize the clinical and biological basis of skin toxicities in immunoradiotherapy and highlight data-driven approaches that aim to shift toxicity care from reactive grading to anticipatory, precision management (**Figure 1**).

## 2 Clinical spectrum of cutaneous toxicities in immunoradiotherapy

### 2.1 Radiation-induced skin injury

Radiation-induced skin injury remains one of the most common toxicities encountered during external beam radiotherapy (15). Its clinical manifestations range from mild erythema and dry desquamation to moist desquamation and ulceration (16). In routine practice, patients receiving radiotherapy to the head and neck, breast, or pelvic regions frequently develop visible skin reactions within the first few weeks of treatment, even under modern conformal techniques (17, 18). Notably, the severity and progression of dermatitis often vary markedly among patients treated with comparable dose–fractionation schemes, highlighting the limited predictive value of physical dose metrics alone (19, 20).

Beyond local tissue damage, radiation-induced skin injury initiates inflammatory signaling, disrupts epidermal barrier function, and reshapes the local immune microenvironment (21–24). These radiation-altered skin sites may persist as zones of heightened sensitivity, creating a biological context in which subsequent immune activation can provoke exaggerated or prolonged cutaneous responses. Clinically, radiation dermatitis is typically field-restricted and temporally linked to local exposure, which enables plan-aware monitoring and mitigation.

### 2.2 Immune-related cutaneous adverse events

Cutaneous toxicities are the most frequently reported immune-related adverse events associated with immune checkpoint inhibitors (25). Clinically, patients may present with diffuse pruritic eruptions, psoriasiform plaques, or lichenoid dermatitis that develop independently of radiation fields and may recur intermittently during treatment (26, 27). Although many of these reactions are manageable with topical therapy, their unpredictable onset and fluctuating course complicate long-term treatment planning (28).

Importantly, immune-related cutaneous adverse events do not follow clear dose–response relationships and may emerge after variable latency periods (29). In some patients, mild early skin manifestations precede more extensive inflammatory reactions, whereas others remain asymptomatic despite prolonged immune exposure, underscoring substantial inter-individual heterogeneity (30). Compared with classic radiation dermatitis, immune-related eruptions more often extend beyond irradiated fields and may show greater responsiveness to immunosuppressive or immunomodulatory management.

### 2.3 Cutaneous toxicity in combined immunoradiotherapy: additive or synergistic effects?

In the setting of combined immunoradiotherapy, cutaneous toxicities often display hybrid features that defy conventional classification (24). A commonly observed clinical scenario involves immune-related skin eruptions preferentially arising within or adjacent to previously irradiated areas, even when immunotherapy is initiated weeks to months after radiotherapy completion (7, 31, 32). Such patterns suggest that radiation-induced tissue remodeling may prime local immune responses, lowering the threshold for subsequent inflammatory injury (22). A radiation-conditioned field may therefore act as a permissive substrate for checkpoint blockade–driven immune amplification, producing field-restricted exacerbation, prolonged courses, or recall-like flares.

From a clinical perspective, these atypical presentations challenge existing toxicity grading systems and complicate early intervention decisions (33, 34). The inability to prospectively identify patients at risk for clinically significant skin toxicity remains a major unmet need, reinforcing the rationale for integrative, data-driven predictive approaches in immunoradiotherapy. Risk modeling in this setting benefits from incorporating spatial dose topology, baseline tissue susceptibility, and systemic immune activation signals rather than relying on dose magnitude alone.

## 3 Biological basis of cutaneous toxicity in immunoradiotherapy

### 3.1 DNA damage response and oxidative stress: a “danger signal” substrate for inflammation

Radiotherapy initiates cutaneous toxicity by inducing DNA double-strand breaks and oxidative stress in keratinocytes, endothelial cells, and dermal fibroblasts (17). Activation of the DNA damage response (DDR) (e.g., ATM/ATR–CHK1/CHK2 signaling) together with mitochondrial and NADPH oxidase–derived reactive oxygen species (ROS) can promote cell stress and death programs, which may amplify the release of endogenous danger signals (35, 36). These damage-associated molecular patterns (DAMPs), including extracellular ATP and alarmins, translate local tissue injury into an inflammatory milieu that primes subsequent immune activation. Clinically, this DDR/ROS axis is relevant because it creates a time-dependent “sensitized window” in irradiated skin during which additional immune stimulation—such as checkpoint blockade—may lower the threshold for overt dermatitis.

### 3.2 Innate immune sensing: cGAS–STING and inflammasome signaling as early amplifiers

A central bridge between radiation injury and immune activation is innate nucleic acid sensing (37). Radiation-induced cytosolic DNA accumulation may activate the cGAS–STING pathway, driving type I interferon programs and chemokine induction that recruit immune cells to damaged skin (38). In parallel, DAMPs such as extracellular ATP and ion flux perturbations can promote inflammasome activation (e.g., NLRP3), leading to IL-1β/IL-18 release and a feed-forward inflammatory loop (39, 40). These innate pathways provide a mechanistic basis for why the same radiation dose can yield different cutaneous outcomes across patients: the magnitude and persistence of innate sensing depend on baseline skin immunity, microbial cues, and host genetic/epigenetic context. Importantly, both type I interferon–dominated responses and IL-1–centered inflammation can potentiate checkpoint inhibitor–driven T-cell activation, potentially favoring additive or synergistic skin toxicity under immunoradiotherapy.

### 3.3 Cytokine and barrier axis: NF-κB/STAT signaling, IL-6–JAK/STAT3, and epidermal integrity

Cutaneous toxicity severity is shaped by how inflammatory signaling intersects with epidermal barrier function (21). Radiation injury activates stress and inflammatory transcriptional programs, prominently NF-κB, which coordinates cytokine and chemokine production (21). Downstream, the IL-6–JAK/STAT3 axis can sustain inflammatory cell recruitment and impair orderly tissue repair, while TNF-α and IL-1 family cytokines further amplify keratinocyte activation and vascular permeability (41). In immune checkpoint blockade, heightened T-cell activity and cytokine release can shift the balance from controlled repair to persistent dermatitis-like inflammation (6). From a translational perspective, this cytokine–barrier axis suggests measurable correlates (e.g., systemic inflammatory markers, local cytokine signatures, barrier disruption phenotypes) that may be integrated into predictive models for toxicity risk stratification.

### 3.4 T-cell effector programs and loss of peripheral tolerance: Th1/Th17 bias and tissue-homing signals

Immune-related skin toxicities are often driven by dysregulated T-cell programs that resemble autoimmune dermatoses. Checkpoint blockade can enhance Th1-skewed IFN-γ signaling and, in some contexts, Th17/IL-17–associated inflammation, promoting keratinocyte activation and neutrophil-linked tissue injury (42). Tissue-homing and retention signals (e.g., chemokine–receptor interactions and resident memory T-cell dynamics) may explain why eruptions can localize to or recur within previously irradiated regions (43). Under immunoradiotherapy, radiation-conditioned skin may display altered chemokine landscapes and antigen exposure, thereby shaping T-cell trafficking and effector persistence (21). This provides a mechanistic rationale for clinically observed patterns such as field-restricted exacerbation and delayed flare phenomena after initiating immunotherapy.

### 3.5 Why pathway knowledge still does not yield reliable patient-level prediction

These pathways—DDR/ROS stress, cGAS–STING and inflammasome signaling, NF-κB/STAT-linked cytokine circuits, and T-cell effector/tolerance disruption—collectively illustrate that cutaneous toxicity in immunoradiotherapy is multi-causal and dynamically evolving. However, pathway activation is rarely observable through a single biomarker, and the clinical phenotype reflects interactions among dose topology, timing, host immunity, and tissue state. Therefore, mechanism-guided hypotheses need to be coupled with integrative, data-driven modeling to achieve actionable, individualized risk prediction—providing a clear rationale for AI-enabled precision toxicity management.

## 4 AI-driven and pathway-informed precision approaches for predicting and managing cutaneous toxicities in immunoradiotherapy

### 4.1 A pathway-to-prediction logic: why mechanistic anchoring matters

Center-to-center variation in schedules, dose geometry, and immune backbones makes purely statistical models prone to poor transportability in immunoradiotherapy (44, 45). Mechanistic anchoring via pathway priors improves plausibility and supports generalization by tying predictors to conserved injury–inflammation programs rather than local practice patterns (46). Pathway-informed modeling is therefore framed as “surrogate-driven” rather than “omics-dependent,” using measurable proxies of damage sensing, interferon tone, inflammasome activity, and cytokine gain (47, 48). This creates a rational bridge between mechanistic heterogeneity and actionable risk stratification.

### 4.2 Damage sensing and innate immune activation: cGAS–STING and type I interferon programs as modelable toxicity drivers

Radiation-associated cytosolic DNA can trigger cGAS–STING signaling, shaping type I interferon programs and chemokine gradients that sustain immune recruitment in irradiated skin (49). When checkpoint blockade is present, an IFN-centered milieu may amplify antigen presentation and effector recruitment, thereby lowering the clinical threshold for dermatitis (50). Operationalization requires mapping the pathway to observable inputs—peripheral interferon-associated signatures, circulating chemokines, and longitudinal inflammatory trajectories—then coupling these signals with dose topology (51). Models that explicitly capture an “IFN-high” inflammatory state may better identify patients in whom modest skin hotspots precipitate disproportionate reactions, thereby enabling earlier prophylaxis and closer monitoring (5).

### 4.3 Inflammasome and IL-1 family signaling: NLRP3-centered amplification and therapeutic decision support

In parallel with interferon programs, radiation injury and DAMP release can activate inflammasome pathways such as NLRP3, leading to IL-1β/IL-18 secretion and a feed-forward inflammatory loop that sustains erythema, edema, and barrier disruption (52). This axis offers a compact explanation for trajectories that accelerate despite stable treatment parameters, especially when checkpoint blockade heightens inflammatory responsiveness (7). Modeling can therefore emphasize features reflecting myeloid activation and systemic inflammatory tone and use risk stratification to prioritize earlier escalation of anti-inflammatory supportive care (21, 53).

### 4.4 Cytokine amplification circuits: NF-κB and IL-6–JAK/STAT3 as bridges from local injury to systemic inflammatory gain

Cutaneous toxicity severity is often governed by cytokine amplification rather than by initial injury magnitude alone (54). Radiation activates NF-κB–linked transcriptional inflammation, while IL-6–JAK/STAT3 signaling sustains leukocyte recruitment, delays repair, and stabilizes inflammatory states (21). Checkpoint blockade can further amplify these circuits by enhancing effector cytokine production and reducing peripheral tolerance (51). For prediction, these circuits are attractive because they are proxied by routine acute-phase reactants and longitudinal blood indices, enabling fusion with dose and imaging features without requiring deep profiling. Clinically, recognizing a “cytokine-amplified” state can guide supportive-care timing, surveillance intensity, and plan-aware mitigation when modifiable hotspots exist (15).

### 4.5 Effector T-cell programs and tissue homing: Th1/Th17-biased inflammation and field-restricted flare patterns

Immune-mediated cutaneous toxicity commonly reflects dysregulated effector programs, including Th1/IFN-γ polarization and, in subsets, Th17/IL-17 activity linked to neutrophil-associated injury (42). Under immunoradiotherapy, radiation-conditioned skin may exhibit altered chemokine landscapes and antigen exposure that facilitate tissue-homing and resident memory dynamics, offering a mechanistic rationale for field-restricted exacerbations or flare phenomena in previously irradiated regions (31). Model design can mirror this biology by combining spatial descriptors (dose geometry and imaging features capturing the “field substrate”) with systemic immune surrogates indexing sustained effector activation (55). Such fusion is aligned with the precision objective of distinguishing patients who will experience limited, self-limited erythema from those at risk of persistent, treatment-disrupting dermatitis (56).

### 4.6 Multi-modal fusion: coupling dose topology, tissue susceptibility, and pathway state

A pragmatic precision framework couples three interacting domains: exposure geometry (dose topology), tissue susceptibility (imaging-/radiomics-derived vulnerability and barrier proxies), and systemic immune gain (pathway-indexing biomarkers) (50). Radiomics can quantify baseline vulnerability and early subclinical change, while pathway-aligned blood features provide a mechanistic readout of interferon, inflammasome, and cytokine amplification states (57). Learning cross-domain interactions renders previously qualitative observations quantifiable—for example, why near-identical dosimetry produces divergent trajectories under checkpoint blockade, or why reactions cluster within particular field geometries in a subset of patients (55, 58). Importantly, this approach allows models to remain actionable even when multi-omics is not universally available, because pathway state can be approximated through accessible clinical surrogates and longitudinal trends.

### 4.7 Clinical applications: from prediction to anticipatory intervention and adaptive management

The value of pathway-informed AI is maximized when linked to specific clinical actions rather than to retrospective grading. Pre-treatment models can identify patients with a high likelihood of interferon- or cytokine-amplified responses who may benefit from intensified prophylactic skincare, tighter follow-up schedules, and plan-aware hotspot minimization when feasible (59). On treatment, dynamic updating using early imaging or symptom trajectories can detect acceleration before thresholds are crossed, prompting timely escalation of topical/systemic management and reducing avoidable interruptions (60). More broadly, by framing cutaneous toxicity as a precision-managed endpoint of immunoradiotherapy, pathway-anchored models can support individualized risk–benefit discussions and rationalize supportive-care resource allocation, thereby translating “data and precision” into concrete patient-level benefit.

## 5 Clinical translation: from prediction to precision toxicity management in immunoradiotherapy

Clinical translation is judged less by retrospective accuracy than by whether model outputs can be embedded into real-world immunoradiotherapy workflows to move practice from reactive grading to anticipatory management (61). Cutaneous toxicity is a particularly compelling entry point for precision approaches because it is common, emerges early, and can be followed longitudinally, yet it is simultaneously governed by interacting determinants spanning dose topology, tissue susceptibility, and systemic immune amplification (34). Accordingly, deployable strategies should begin with multimodal risk stratification that integrates clinicodosimetric variables with spatial dose descriptors and imaging phenotypes that represent the local injury substrate, while incorporating accessible inflammatory and immune surrogates that approximate pathway states such as interferon-dominant activation, IL-1–centered amplification, or cytokine-driven persistence (62). Such biological anchoring strengthens interpretability and supports clinically actionable stratification rather than purely correlational labeling.

Before treatment initiation, prediction only becomes meaningful when it maps to modifiable decision points. At the planning stage, risk information should redirect attention from coarse summary metrics to actionable features of exposure geometry, particularly skin-surface hotspots and steep gradients in high-risk regions, and prioritize feasible re-optimization without compromising target coverage (63). In immunoradiotherapy, modest adjustments to exposure structure may yield disproportionate benefit in patients whose systemic inflammatory “gain” is predicted to be high (64). In parallel, supportive care need not be uniform; stratified pathways can justify intensified prophylactic barrier protection and closer surveillance for patients flagged as immune-amplified responders, while emphasizing local exposure mitigation and context factors such as friction and moisture for those whose risk appears predominantly exposure-driven (34). The goal is not overtreatment but rational allocation of preventive intensity to the patients most likely to avoid escalation and treatment disruption.

During therapy, the highest-impact application is dynamic updating rather than a single baseline score (65). Cutaneous toxicity follows recognizable temporal kinetics, and the actionable signal is often an inflection toward accelerating inflammation rather than a late-stage grade change. Incorporating longitudinal symptom trajectories and standardized skin assessments—optionally augmented by imaging- or photograph-derived quantitative signals—allows models to detect early acceleration within the intervention window and trigger timely escalation of management to reduce interruptions and dose compromise (66). For adoption, performance metrics must be paired with external validation, calibration to actionable thresholds, and resilience to evolving immunotherapy backbones. Ultimately, utility should be judged by hard clinical endpoints—reductions in intervention-requiring dermatitis, unscheduled visits, and treatment discontinuation—thereby establishing cutaneous toxicity as a precision-managed component of immunoradiotherapy delivery.

## 6 Discussion

Several barriers currently limit the reliable translation of AI-enabled prediction into routine immunoradiotherapy care. A central issue is endpoint heterogeneity: cutaneous toxicities are typically captured by ordinal grading systems that are partly subjective and vary across clinicians and centers, while “clinically meaningful” outcomes differ (any-grade events versus intervention-requiring dermatitis, treatment interruption, or persistent toxicity) (67). Without harmonized definitions and robust phenotyping, models may appear performant yet fail to generalize or trigger appropriate actions. Future work should standardize toxicity endpoints, incorporate patient-reported outcomes to reflect symptom burden, and explicitly align modeling targets with actionable clinical decisions.

Data fragmentation and domain shift further constrain generalizability. Immunoradiotherapy backbones, sequencing strategies, and concomitant therapies vary widely, and radiotherapy planning and imaging protocols introduce center-specific signatures that can confound learning, particularly for radiomics (63). Uneven representation of subgroups—such as different skin types, anatomical sites, and moisture/friction-prone regions—can bias risk estimates. Addressing these issues will require multicenter prospective datasets, rigorous external validation, calibration of actionable thresholds, and evaluation of subgroup performance, with domain adaptation strategies when necessary.

A final challenge is limited biological observability and mechanistic drift over time. Although pathway anchoring improves plausibility, key axes such as interferon programs, inflammasome activation, and cytokine amplification are rarely measured directly, forcing reliance on imperfect proxies that may change under treatment (63). Priority directions include developing minimal, clinically feasible biomarker panels, integrating longitudinal sampling to capture temporal dynamics, and testing model utility in pragmatic prospective studies that evaluate reductions in intervention-requiring dermatitis and treatment disruption (66, 68). Standardized smartphone photography with automated quantification may provide scalable longitudinal inputs for real-time risk updating and workflow-integrated management.
